# Prevalence and Risk Factors of Hepatocellular Carcinoma in Patients Co-infected With Hepatitis B Virus (HBV) and Hepatitis C Virus (HCV): A Cross-Sectional Retrospective Study

**DOI:** 10.7759/cureus.85985

**Published:** 2025-06-14

**Authors:** Saleem Iqbal, Zarak Qureshi, Hafiz Muhammad Mudasir, Asif Khan, Amir Sohail, Jamil Ahmad, Fatima Tu Zuhra, Muhammad Younas Ali, Moosa Ali, Zubair Ahmad, Kainat Khan

**Affiliations:** 1 General Medicine, Hayatabad Medical Complex, Peshawar, PAK; 2 Acute Internal Medicine, Blackpool Teaching Hospitals NHS Foundation Trust, Blackpool, GBR; 3 General Medicine, Mufti Mehmood Memorial Teaching Hospital, Dera Ismail Khan, PAK; 4 Internal Medicine, Hayatabad Medical Complex, Peshawar, PAK; 5 Internal Medicine, Khyber Teaching Hospital, Peshawar, PAK; 6 Internal Medicine, Provincial Headquarter Teaching Hospital, Gilgit, PAK; 7 General Medicine, Mayo University Hospital, Castlebar, IRL; 8 Community Medicine, Khyber Medical College, Peshawar, PAK; 9 Internal Medicine, Lady Reading Hospital, Peshawar, PAK

**Keywords:** afp, cirrhosis, hbv-hcv co-infection, hepatocellular carcinoma, pakistan, risk factors

## Abstract

Background

A serious consequence of long-term hepatitis B virus (HBV) and hepatitis C virus (HCV) infections is hepatocellular carcinoma (HCC), particularly in co-infected individuals, who are at a significantly higher risk of malignant transformation.

Objective

The main objective of this study is to determine the frequency and identify associated risk factors of HCC in patients co-infected with HBV and HCV through a retrospective cross-sectional analysis.

Methodology

This retrospective cross-sectional study was conducted at Hayatabad Medical Complex, Peshawar, Pakistan, using medical records of HBV-HCV co-infected patients from January 2023 to December 2024. A total of 348 patients aged ≥18 years, with complete clinical, biochemical, and radiological/histopathological data, were included. Data were analyzed using IBM SPSS Statistics for Windows, Version 25 (Released 2017; IBM Corp., Armonk, NY, USA), evaluating demographics, comorbidities, alpha-fetoprotein (AFP) levels, liver function tests, and HCC diagnosis. Chi-square and logistic regression analyses were used to identify significant risk factors, with p-values <0.05 considered statistically significant.

Results

Out of 348 HBV-HCV co-infected patients, 73 (20.98%) were diagnosed with HCC. The majority of patients were aged between 31 and 60 years, with a mean age of 47.8 ± 12.6 years. Males comprised 58.91% (n = 205) of the cohort. Among the HCC patients, age >45 years was significantly associated with HCC occurrence (71.23% vs. 47.27%, p = 0.002). Cirrhosis was found in 68.49% of HCC patients versus 35.64% without HCC (p < 0.001). Infection duration ≥5 years was observed in 72.60% of HCC cases compared to 60.36% of non-HCC cases (p = 0.054). Elevated AFP >20 ng/mL was significantly associated with HCC (61.64% vs. 24.36%, p < 0.001), as was elevated alanine aminotransferase (ALT) (76.71% vs. 57.82%, p = 0.006). Comorbidities were more common in HCC patients (39.73%) compared to non-HCC patients (21.82%, p = 0.004). Logistic regression analysis identified several independent predictors of HCC: age >45 years (adjusted odds ratio (AOR): 2.45; 95% CI: 1.38-4.36; p = 0.002), cirrhosis (AOR: 3.87; 95% CI: 2.15-6.96; p < 0.001), infection duration ≥5 years (AOR: 1.62; 95% CI: 1.01-2.59; p = 0.046), AFP >20 ng/mL (AOR: 3.09; 95% CI: 1.74-5.48; p < 0.001), and presence of comorbidities (AOR: 1.85; 95% CI: 1.09-3.15; p = 0.022).

Conclusion

Among HBV-HCV co-infected patients, a high proportion of HCC was observed. Significant risk factors independently associated with HCC included age over 45 years, presence of cirrhosis, longer infection duration, elevated AFP levels, and comorbidities. These findings underscore the importance of early risk stratification and regular surveillance in co-infected individuals to enable timely diagnosis and intervention.

## Introduction

The most prevalent primary liver cancer, and one of the main causes of cancer-related mortality worldwide, is hepatocellular carcinoma (HCC) [1]. Particularly in areas where viral hepatitis is more prevalent, the incidence of HCC is still increasing [2]. The pathophysiology of HCC is known to be significantly influenced by chronic infection with the hepatitis B virus (HBV) and hepatitis C virus (HCV) [3]. The individual functions of HBV and HCV in liver carcinogenesis have been well-established by study; however, it is still unclear what the clinical ramifications and carcinogenic synergy of co-infection are [4].

Compared to patients with mono-infection, patients with co-infections of HBV and HCV have a more complicated clinical picture, often showing greater rates of cirrhosis, faster liver disease development, and an elevated risk of hepatocarcinogenesis [5]. Hepatic fibrosis, immune-mediated damage, and chronic inflammation may all be brought on by the simultaneous viral attack on hepatocytes, which together may promote malignant transformation [6]. Furthermore, a physiologically hostile environment that may increase the risk of HCC is produced by the combination of HBV DNA incorporation into the host genome and the long-term cytopathic effects of HCV [7].

The epidemiological patterns of HBV-HCV co-infection and related HCC development are also influenced by geographic and population-based variations [8,9]. Poor monitoring, delayed diagnosis, and restricted access to antiviral medication all lead to late-stage HCC discovery and worse clinical outcomes in many low- and middle-income nations, including Pakistan [10]. Although the issue is becoming more widely acknowledged, there is still a lack of thorough, geographically specific data on the prevalence of HCC and the risk factors linked to it in co-infected people [11]. In order to guide treatment approaches, inform screening measures, and eventually enhance survival outcomes, this gap must be filled.

In HBV-HCV co-infected populations, the interaction of virus, host, and environmental variables has to be thoroughly studied. This research aims to clarify important demographic, clinical, and biochemical risk variables that contribute to disease progression by assessing the burden of HCC in patients with HBV-HCV co-infection using a cross-sectional, retrospective approach.

Research objective

The main objective is to determine the incidence and identify the associated risk factors of HCC in patients co-infected with HBV and HCV, through a retrospective, cross-sectional analysis.

## Materials and methods

Study design and setting

This cross-sectional, retrospective study was conducted at Hayatabad Medical Complex, Peshawar, Pakistan, a major tertiary care hospital that provides specialized liver disease services. The study involved the systematic review of medical records for patients co-infected with HBV and HCV between January 2023 and December 2024. This timeframe was chosen to ensure an adequate sample size and the availability of recent and complete clinical data. The study design allowed for the identification of HCC among co-infected individuals and the assessment of multiple associated risk factors.

Inclusion and exclusion criteria

Patients were included if they met all of the following criteria: aged 18 years or older, confirmed diagnosis of both HBV and HCV, availability of complete medical records, and undergoing radiological and/or histological investigations for HCC within the study period.

Exclusion criteria involved a history of other chronic liver disorders, such as alcoholic liver disease, Wilson’s disease, and autoimmune hepatitis, or incomplete clinical documentation that hindered HCC diagnosis or evaluation. These criteria ensured that only reliable and relevant cases were analyzed for risk assessment.

Sample size

A total of 348 patient records meeting the above inclusion criteria were selected through convenience sampling. This method involved retrieving cases directly from hospital records without randomization. Among these patients, 73 (21%) were diagnosed with HCC, and 275 (79%) were not, forming two comparison groups for evaluating potential predictors of HCC development in HBV-HCV co-infected individuals.

Data collection

A standardized data collection form was used to retrieve data from both paper-based and electronic hospital records (see Appendices). Extracted variables included demographic data (age and sex); clinical history (duration of HBV and HCV infection, presence of cirrhosis, and comorbidities); laboratory data (alanine aminotransferase (ALT), aspartate aminotransferase (AST), and alpha-fetoprotein (AFP) levels); and radiological and histological assessments (ultrasound and computed tomography/magnetic resonance imaging (CT/MRI) findings). Duration of infection was calculated based on the earliest recorded date of HBV or HCV diagnosis in the patient's medical file, using serological or polymerase chain reaction (PCR)-confirmed laboratory results. If both viruses were diagnosed at different times, the longer of the two durations was considered. This approach provided a conservative estimate of infection duration, suitable for risk factor analysis.

Statistical analysis

Data were analyzed using IBM SPSS Statistics for Windows, Version 25 (Released 2017; IBM Corp., Armonk, NY, USA). Descriptive statistics summarized demographic, clinical, and laboratory variables. The Chi-square test was employed to examine associations between categorical variables and HCC status. Variables with p-values <0.05 in univariate analyses were included in a multivariate logistic regression model to identify independent predictors of HCC, thereby adjusting for potential confounding factors.

Ethical approval

The Hayatabad Medical Complex Hospital Research and Ethical Committee granted ethical permission for the research (Approval No. 319/HMC/2022, dated July 22, 2022). Data were utilized only for research purposes, and patient confidentiality was upheld.

## Results

The demographics of 348 HBV-HCV co-infected patients are shown in Table 1, which reveals that most of the patients were between the ages of 31 and 45 (112 patients, or 32.18%) and 46 and 60 (109 patients, or 31.32%), with fewer patients over 60 (73 patients, or 20.98%) and between 18 and 30 (54 patients, or 15.52%). Regarding sex distribution, 205 patients (58.91%) were male, and 143 patients (41.09%) were female.

**Table 1:** Demographic Characteristics of HBV-HCV Co-infected Patients (n = 348) HBV-HCV, hepatitis B virus and hepatitis C virus

Category	Characteristic	Number (%)
Age Group	18-30	54 (15.52%)
	31-45	112 (32.18%)
	46-60	109 (31.32%)
	>60	73 (20.98%)
Sex	Male	205 (58.91%)
	Female	143 (41.09%)

Clinical and laboratory characteristics are shown in Table 2, where 219 patients (62.93%) had an infection duration of five years or more, whereas 129 patients (37.07%) had an infection duration of less than five years. A total of 148 patients (42.53%) had cirrhosis. Liver enzymes were often increased: 215 patients (61.78%) had raised ALT, and 198 patients (56.70%) had elevated AST. Comorbidities such as diabetes were identified in 89 patients (25.57%), while 112 patients (32.18%) had AFP levels >20 ng/mL, a tumor marker for HCC.

**Table 2:** Clinical and Laboratory Characteristics of Patients (n = 348) ALT, alanine aminotransferase; AST, aspartate aminotransferase; AFP, alpha-fetoprotein

Category	Parameter	Number (%)
Duration of Infection	<5 years	129 (37.07%)
	≥5 years	219 (62.93%)
Presence of Cirrhosis	Yes	148 (42.53%)
	No	200 (57.47%)
Liver Enzymes	Elevated ALT (>40 U/L)	215 (61.78%)
	Elevated AST (>40 U/L)	198 (56.90%)
Tumor Marker	AFP >20 ng/mL	112 (32.18%)
Comorbidities	Present (e.g., diabetes)	89 (25.57%)

**Figure 1:**
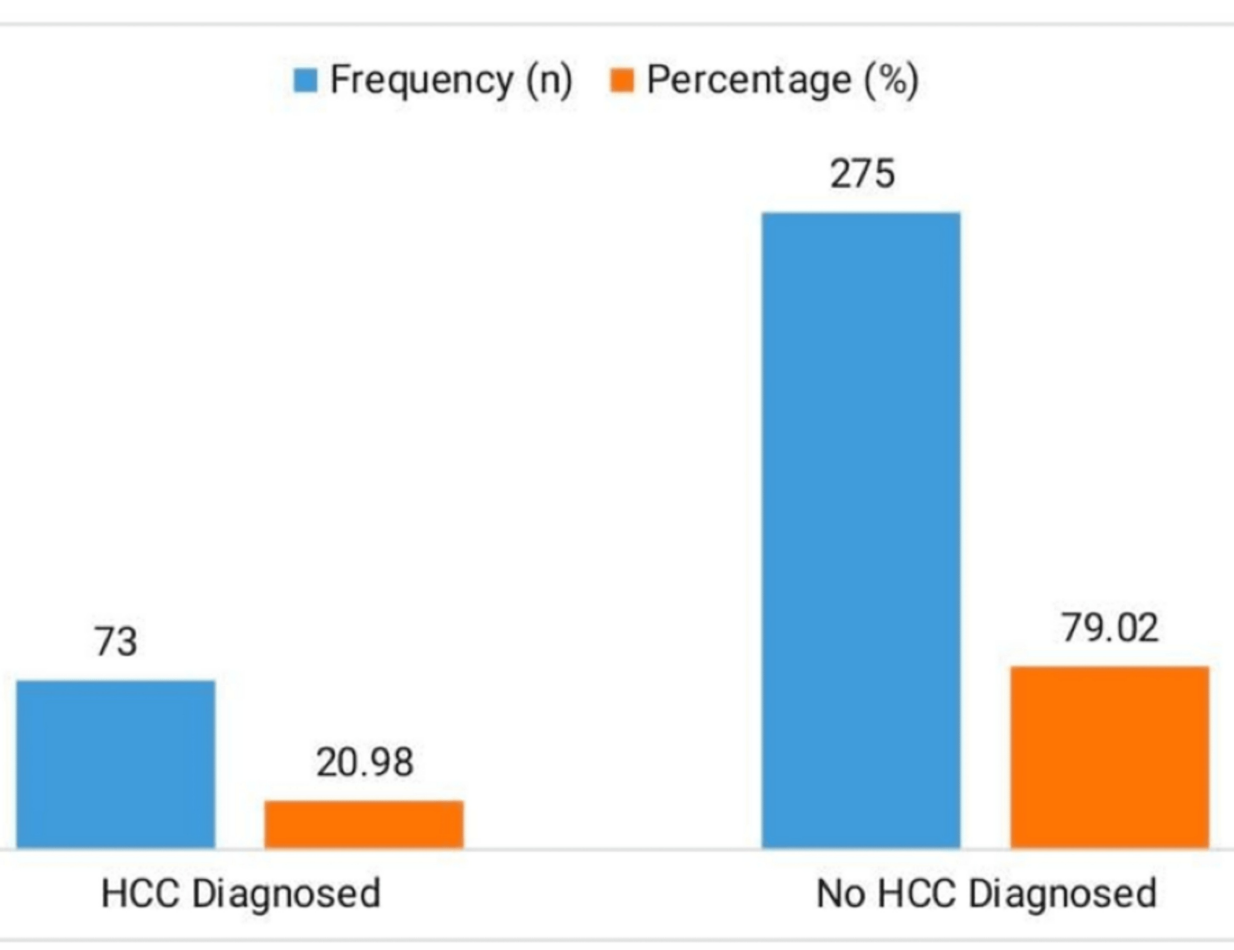
Incidence of HCC Among HBV-HCV Co-infected Patients HCC, hepatocellular carcinoma; HBV-HCV, hepatitis B virus and hepatitis C virus

The cohort's incidence of HCC is shown in Figure 1, where 73 individuals (20.98%) were diagnosed with HCC, and the other 275 patients (79.02%) were not.

When comparing risk factors between patients with HCC (n = 73) and those without HCC (n = 275), Table 3 shows that the proportions of patients with cirrhosis (50, 68.49% vs. 98, 35.64%; p < 0.001), infection duration ≥5 years (53, 72.60% vs. 166, 60.36%; p = 0.054), AFP >20 ng/mL (45, 61.64% vs. 67, 24.36%; p < 0.001), elevated ALT (56, 76.71% vs. 159, 57.82%; p = 0.006), comorbidities (29, 39.73% vs. 60, 21.82%; p = 0.004), and age over 45 years were significantly higher. There was no discernible difference in male patients.

**Table 3:** Risk Factors Associated With HCC Development in HBV-HCV Co-infected Patients (n = 348) Univariate associations are shown. Variables with p < 0.05 were included in the multivariate logistic regression to adjust for confounders. *p < 0.05 was considered statistically significant. HCC, hepatocellular carcinoma; HBV-HCV, hepatitis B virus and hepatitis C virus; ALT, alanine aminotransferase; AFP, alpha-fetoprotein

Risk Factor	HCC Present (n = 73); Number (%)	HCC Absent (n = 275); Number (%)	Chi-square (X²)	p-value*
Age >45 years	52 (71.23%)	130 (47.27%)	13.275	0.002
Male Sex	48 (65.75%)	157 (57.09%)	1.788	0.181
Presence of Cirrhosis	50 (68.49%)	98 (35.64%)	25.48	<0.001
Duration of Infection ≥5 years	53 (72.60%)	166 (60.36%)	3.704	0.054
AFP >20 ng/mL	45 (61.64%)	67 (24.36%)	36.733	<0.001
Elevated ALT	56 (76.71%)	159 (57.82%)	8.722	0.006
Comorbidities Present	29 (39.73%)	60 (21.82%)	9.719	0.004

The findings of the logistic regression identifying independent risk variables for the development of HCC are shown in Table 4. The odds of developing HCC in patients with HBV-HCV co-infection were significantly increased by: age over 45 years (2.45 times; 95% CI: 1.38-4.36, p = 0.002), cirrhosis (3.87 times; 95% CI: 2.15-6.96, p < 0.001), infection duration ≥5 years (1.62 times; 95% CI: 1.01-2.59, p = 0.046), AFP >20 ng/mL (3.09 times; 95% CI: 1.74-5.48, p < 0.001), and comorbidities (1.85 times; 95% CI: 1.09-3.15, p = 0.022).

**Table 4:** Logistic Regression Analysis of Significant Risk Factors for HCC Development Multivariate logistic regression was used to adjust for confounding between variables included in the model. However, some potential confounders, such as antiviral therapy, alcohol use, smoking, and BMI, were not available and thus were not adjusted for. *p < 0.05 was considered statistically significant. HCC, hepatocellular carcinoma; AFP, alpha-fetoprotein; BMI, body mass index

Risk Factor	Adjusted Odds Ratio (AOR)	95% Confidence Interval (CI)	p-value
Age >45 years	2.45	1.38 - 4.36	0.002
Presence of Cirrhosis	3.87	2.15 - 6.96	<0.001
Duration of Infection ≥5 years	1.62	1.01 - 2.59	0.046
AFP >20 ng/mL	3.09	1.74 - 5.48	<0.001
Comorbidities Present	1.85	1.09 - 3.15	0.022

## Discussion

The increased carcinogenic potential of dual viral infection was highlighted by the current study's finding that the incidence of HCC among patients with HBV-HCV co-infection was 20.98% (73 out of 348 patients). This frequency is consistent with research conducted in areas with comparable epidemiological patterns. For example, a prior study found that 14% of co-infected individuals had HCC, indicating a similar prevalence in low- to middle-income nations with inadequate viral hepatitis treatment [12].

Given that 71.23% of HCC patients were over 45, age was shown to be a major risk factor. This finding aligns with scientific knowledge that liver damage and cumulative viral exposure increase with age. Age above 40 was also identified in earlier studies as a significant predictor of HCC in hepatitis patients, linked to genomic instability and increased liver fibrosis over time [13,14]. Age is further supported as an independent risk factor in our analysis by the adjusted odds ratio (AOR) of 2.45 (95% CI: 1.38-4.36; p = 0.002).

With an AOR of 3.87 (95% CI: 2.15-6.96), cirrhosis was seen in 68.49% of HCC patients compared to 35.64% of non-HCC patients (p < 0.001). This is consistent with prior research that found cirrhosis in more than 70% of HCC patients in populations infected with the hepatitis virus [15]. It is well known that the fibrotic environment serves as a prelude to malignant transformation, especially when chronic hepatitis is present.

Another important factor was the length of HBV-HCV infection: 72.60% of HCC patients had a disease duration of ≥5 years (p = 0.045). A prior investigation that found a high correlation between hepatocarcinogenesis and longer viral persistence showed similar findings [16]. Prolonged co-infection may cause gradual hepatic damage, which could explain the elevated risk of cancer.

Although the availability of direct-acting antivirals (DAAs) has significantly improved the prognosis of HCV monoinfection, their role in co-infected patients remains complex. In many low-resource settings, late diagnosis and delayed treatment initiation may limit their preventive impact on HCC development. While our study did not assess treatment history, future research may explore how timely DAA therapy influences long-term outcomes in HBV-HCV co-infected populations.

The independent predictor of AFP levels >20 ng/mL was validated by logistic regression to be substantially more common among HCC patients (61.64% vs. 24.36%; p < 0.001) (AOR: 3.09; 95% CI: 1.74-5.48). Our results confirm the diagnostic value of elevated AFP, which has long been identified as a characteristic of HCC, especially in high-risk populations [17]. Diabetes and other comorbid diseases were also substantially linked to HCC (39.73% in HCC vs. 21.82% in non-HCC; AOR: 1.85; p = 0.022). This is consistent with the findings of other studies that found metabolic impairment to be a co-factor raising the risk of HCC in hepatitis patients [18].

Study strengths and limitations

This study's strength is a well-defined patient cohort and comprehensive data analysis using appropriate statistical methods, which strengthens the validity of the observed associations. However, several limitations must be acknowledged. The retrospective cross-sectional design restricts causal inference and limits assessment of incidence rates. Additionally, convenience sampling may introduce selection bias, potentially favoring sicker patients or those undergoing more extensive investigations. Key variables that could act as significant confounders - such as HBV DNA and HCV RNA viral loads, history of antiviral therapy, and some lifestyle factors, including alcohol consumption and socioeconomic status - were not available in the medical records and therefore were not included in the analysis. The absence of these data limits the depth of risk factor evaluation and may contribute to residual confounding. These limitations should be carefully considered when interpreting the findings.

## Conclusions

The incidence of HCC among HBV-HCV co-infected patients is noteworthy (20.98%), and there are strong correlations between the development of HCC and variables like age over 45, cirrhosis, length of infection, elevated AFP levels, and comorbidities such as diabetes, according to this study. These results highlight the need for improved monitoring, prompt identification, and timely treatment of patients who are co-infected, particularly those with high-risk characteristics. Reducing the burden of HCC in co-infected patients requires region-specific interventions that focus on integrated treatment and early identification.

## Appendices

**Table 5:** Study Questionnaire

Section	Variable/Question	Response Options/Format
Demographics	Patient ID (coded)	__________
Demographics	Age	_______ years
Demographics	Sex	☐ Male ☐ Female
Clinical History	Duration of HBV infection	_______ years
Clinical History	Duration of HCV infection	_______ years
Clinical History	Presence of cirrhosis	☐ Yes ☐ No
Clinical History	Comorbidities present?	☐ Yes ☐ No (If Yes, specify: ________________________)
Laboratory Data	ALT level	_______ U/L
Laboratory Data	AST level	_______ U/L
Laboratory Data	AFP level	_______ ng/mL
Imaging & Histology	Imaging performed for HCC?	☐ Yes ☐ No
Imaging & Histology	Imaging result indicative of HCC?	☐ Yes ☐ No ☐ Not Available
Imaging & Histology	Histological confirmation of HCC?	☐ Yes ☐ No ☐ Not Applicable
Diagnosis	Final diagnosis of HCC	☐ Confirmed ☐ Not Confirmed
Eligibility Checklist	Age ≥ 18	☐ Yes ☐ No
Eligibility Checklist	Diagnosed with both HBV and HCV	☐ Yes ☐ No
Eligibility Checklist	Radiological/histological HCC assessment available	☐ Yes ☐ No
Eligibility Checklist	Any other liver disease (alcoholic liver disease, Wilson’s, autoimmune hepatitis)?	☐ Yes ☐ No
Eligibility Checklist	Sufficient medical record for assessment	☐ Yes ☐ No
Data Source	Medical record type	☐ Electronic ☐ Paper-based
Ethical Consideration	Data used only for research & patient confidentiality is maintained	☐ Yes
